# A platform for multisite immune profiling of premetastatic pancreatic cancer at single-cell resolution

**DOI:** 10.1007/s00262-025-04146-5

**Published:** 2025-08-23

**Authors:** Elishama N. Kanu, Ashley A. Fletcher, Jiayin Bao, Ethan S. Agritelley, Julia Button, Austin M. Eckhoff, Karrie Comatas, Tao Wang, Bin-Jin Hwang, Michael E. Lidsky, Sabino Zani, Dan G. Blazer, Peter J. Allen, Zhicheng Ji, Frank J. Lowery, Sri Krishna, Nicholas D. Klemen, Daniel P. Nussbaum, Erika J. Crosby

**Affiliations:** 1 Department of Surgery, Duke University Medical Center, 2301 Erwin Road, Durham, NC 27701 USA; 2 Duke University School of Medicine, Duke University, Durham, NC USA; 3 Department of Biostatistics and Bioinformatics, Duke University School of Medicine, Duke University, Durham, NC USA; 4 Surgery Branch, Center for Cancer Research, National Cancer Institute, National Institutes of Health, Bethesda, MD USA

**Keywords:** Single cell RNAseq, PDAC, Pre-metastatic liver, TCR, T cell clonality

## Abstract

**Background:**

Pancreatic ductal adenocarcinoma (PDAC) is characterized by exceedingly high rates of metastatic progression, with the liver representing the most common site of distant spread. Here, we established a platform for multisite immune profiling of human PDAC encompassing the tumor, peripheral circulation, and premetastatic liver, to more comprehensively study how various immune subsets might contribute to patient outcomes.

**Methods:**

Tumor, liver, and blood samples were obtained from patients undergoing resection for non-metastatic PDAC. Derived immune cells underwent paired single-cell RNA and TCR sequencing. Immune composition, cell-type functional profiles, and T cell clonal expansion patterns were evaluated across tissue sites.

**Results:**

In total, 106,539 immune cells were sequenced, of which 85,748 met criteria for analysis. We identified 32 cell populations, of which seven demonstrated significant enrichment within a particular tissue, highlighting that this workflow possesses the granularity needed for identifying potential future biomarkers. Functional profiling revealed tissue-specific differences in cell phenotypes. This included terminally differentiated exhausted CD8 T cells within the tumor, highly active Tregs within the premetastatic liver and tumor, and M1 versus M2 polarization of liver and tumor macrophage populations, respectively. Within the tumor, expanded Treg clones were uniquely abundant, and while expanded clones could be tracked to the blood and premetastatic liver, many of these mapped back to known viral antigens. Leveraging previously validated gene sets, we show how these can be applied to predict the tumor reactivity of intratumoral T cells using transcriptional signatures. We demonstrated a high degree of concordance between multiple independent signatures and tracked high-priority TCRs within the blood and liver.

**Conclusion:**

This study demonstrates the feasibility of a platform, which has already been implemented into ongoing clinical protocols, for immune profiling of human PDAC across the sites most relevant to metastatic progression. Future applications of this work can monitor immune populations throughout metastatic progression to build a temporal database of immune phenotypes and track association with clinical outcomes.

**Supplementary Information:**

The online version contains supplementary material available at 10.1007/s00262-025-04146-5.

## Introduction

Pancreatic ductal adenocarcinoma (PDAC) is typically an incurable disease, characterized either by advanced stage at diagnosis or by recurrence and metastatic progression in patients who initially present with a localized primary tumor [1, 2]. The liver is the most common site of metastatic spread, and patients with hepatic metastases have the worst survival outcomes, including patients with multisite disease [2, 3]. Therefore, there is a critical need to advance therapeutic strategies with the potential to prevent and target liver metastases, which first requires an improvement in our fundamental understanding of the processes driving hepatic progression.

The formation of a “premetastatic niche,” whereby a distant organ is primed for metastatic dissemination, has been proposed to explain the preferential homing of metastatic cells within specific sites [4, 5]. However, the mechanisms driving these changes within the liver remain nebulous [6, 7]. At baseline, the liver exhibits immunosuppressive properties, evidenced for example by immune tolerance during organ transplantation even in the absence of systemic immune suppression [8, 9]. In the setting of cancer, this may be further exacerbated through remodeling of the hepatic immune landscape by recruitment of myeloid cells and other immunosuppressive populations [10–12]. Recent advances in high-throughput single-cell sequencing now allow for exploration of the role of individual cell types and their interactions. Still, investigation of premetastatic hepatic immunity in human PDAC remains largely unstudied, due chiefly to the lack of appropriate tissue availability (i.e., “normal” liver samples in patients at high risk for metastatic spread). Thus, our limited understanding of premetastatic liver immunity comes almost exclusively from animal models [13–15].

To address this deficit, we developed a unique biobanking effort to obtain these necessary tissue samples from PDAC patients without detectable metastatic disease. Leveraging this biorepository, we devised a pilot study to establish a pipeline to profile immunity at single-cell resolution across the primary tumor, the peripheral blood, and the premetastatic liver. With an intentionally designed small sample size, we tested the feasibility of this platform to define the immune composition and functionality observed across the multisite premetastatic environment. Herein, we introduce our pipeline as a novel platform to investigate phenotypical and functional differences between tissue cell types, explore patterns of clonal expansion, and apply validated gene signatures to predict potential neoantigen-specific, tumor-reactive T cells from primary PDAC tumors [16–18]. This represents a reproducible platform that can be applied to existing [19] and future larger-scale clinical trials beyond just PDAC, including protocols to generate personalized cellular therapies for metastatic patients.

## Materials and methods

### Patient enrollment

Patients with localized (non-metastatic) PDAC who were treatment-naïve and planning to undergo curative-intent surgical resection were eligible for this study. Included patients were enrolled in the Duke Gastrointestinal and Hepatopancreaticobiliary (GHPB) Biorepository, which is an IRB-approved (Pro00108288) tissue acquisition protocol for patients with various gastrointestinal malignancies. Informed consent was obtained for all patients.

### Sample collection

Biospecimens from three different sites were acquired for this study: peripheral blood, primary tumor, and normal liver. Peripheral blood collection was performed just prior to surgery, and two 10-mL acid citrate dextrose (ACD) phlebotomy tubes (BD Vacutainer) were collected. Liver samples were obtained immediately following the start of surgery, once the absence of gross metastatic disease had been confirmed. Three approximately 1 cm^3^ biopsies were obtained from separate hepatic segments and placed in RPMI on ice. The primary tumor was obtained following resection and standard pathologic processing, at which point a representative specimen was provided and placed in RPMI on ice.

### Processing of blood samples

Blood samples underwent standard Ficoll separation and centrifugation (760 RCF for 20 min) to isolate the peripheral blood mononuclear cell (PBMC) layer. Isolated PBMCs were centrifuged (274 RCF for 10 min) and resuspended in Dulbecco’s phosphate-buffered saline without added calcium or magnesium (DPBS, Gibco 14190144).

### Processing of liver and primary tumor samples

Tissues were processed within 10 min of receipt to isolate live immune cells. Each specimen was minced and placed in an enzymatic mix of 75 mg/mL collagenase (Sigma-Aldrich C2139), 150 mg/mL dispase II (Sigma-Aldrich D4693), 10 U/μL DNAse (Sigma-Aldrich 11284932001), and 5 mM calcium chloride (Sigma-Aldrich 21115), followed by incubation at 37 °C on a mechanical rocker for 30 min. The digested sample was then filtered through a 70-µM cell strainer, centrifuged (100 RCF for 10 min), and resuspended in red blood cell (RBC) lysis buffer (BioLegend 420301) for ten minutes. The suspension was then filtered through a 40-µM cell strainer, centrifuged (800 RCF for 3 min), and resuspended in phosphate-buffered saline (PBS) with 2.5% fetal bovine serum (FBS) to obtain a single-cell suspension.

### Single-cell sequencing

The liver, tumor, and PBMC single-cell suspensions were stained with anti-human CD45 antibody (0.05 μg/μL FITC; Clone: HI30; eBioscience 2705432) and fixable far red live/dead cell stain (ThermoFisher L10120). Cells were gated on live, single, CD45 + and sorted into 1% BSA-DPBS using a JSAN cell sorter (Bay Bioscience, Japan). For each individual sample, up to 10,000 sorted live, CD45 + cells were loaded onto the Chromium Next GEM Chip K per the 10 × Genomics Single-Cell 5′ Reagent Kits v2 protocol (CG000331 Rev D). Gene expression (GEX) and TCR libraries were generated according to the 10 × protocol. Briefly, after droplet generation, cDNA was generated by reverse transcription, purified using Silane DynaBead clean-up, and then amplified via PCR for sixteen cycles. Paired 5′ GEX and TCR libraries for each sample were constructed from the amplified cDNA and sequenced on the NovaSeq (Illumina) platform at a depth of 50,000 reads per cell for the GEX libraries and 5000 reads per cell with dual indexing for the TCR libraries. All data generated from this study are available at the following database: https://github.com/afletch00/Kanu_Liver_scRNA.git.

### Data integration and cluster annotation

FASTQ files containing the 10 × Chromium scRNA-seq data were processed in CellRanger, and RStudio (Seurat package v4.3.01) was used to filter, analyze, and normalize the data. FASTQs were aligned to the Genome Research Consortium human build 38 (GRCh38) reference provided by 10 × Genomics (refdata-gex-GRCh38-2020-A for GEX libraries and refdata-cellranger-vdj-GRCh38-alts-ensembl-54.0.0. for TCR libraries). *PTPRC*-expressing cells with fewer than 500 total reads or 300 gene features and greater than 25,000 reads, 6,000 gene features, or 15% mitochondrial reads were excluded from analysis. Data from all three tissues were then normalized using Seurat’s “SCTransform” function and integrated with RPCS reduction using the “SelectIntegrationFeatures,” “PrepSCTIntegration,” “FindIntegrationAnchors,” and “IntegrateData” functions. Cell clustering was performed using the “FindAllClusters” function in Seurat at a resolution of 0.7 and using 30 principal component dimensions.

### Cell-type annotation

Differential gene expression (DGE) analysis was performed on the integrated data by running the “FindAllMarkers” function from the Seurat v5.1.0 package for R. Each cluster's top differentially expressed genes were used to annotate broad cell types (B cells, T cells, myeloid cells, and NK cells). For more specific cell-type annotation, each broad cell type was extracted from the larger data and reclustered according to the first 30 principal component dimensions at a resolution of 0.5. For B and T cell sub-clusters, B cell receptor and TCR genes were removed before clustering to avoid these genes driving individual cell clusters. Myeloid cells were clustered at a higher resolution of 1.0 to further separate distinct cell populations. We used two complementary approaches to identify genes that were enriched in a specific cluster. We first identified marker genes specific to each cell cluster by calculating the log2 fold change (log2FC) between one cluster of interest versus all other remaining clusters using Seurat’s “FindAllMarkers” function with the Wilcoxon rank-sum test (default parameters). Genes were ranked based on their expression difference, and the top differentially expressed genes were examined (Supplementary Table S1). We also calculated the average expression for each of the 3000 highly variable genes within the clusters to aid in cell-type annotation (Supplementary Table S1). Classification of immune cell subsets was specified from annotation of cluster-specific and highly expressed genes and supported by previously published gene signatures and expression patterns from Zhao et al. [20], Zhang et al. [21], and the scType database [22]. If a specific immune cell-type subset was unable to be identified/matched, the key differentially expressed gene for that cluster was listed in the cluster name.

### Differential gene expression and gene set enrichment analysis

Differentially expressed genes were determined using a threshold of log2fold change > 0.5 and adjusted p values < 0.05. Volcano plots for DGE comparison analysis between cell types were generated using the same threshold criteria. Gene set enrichment analysis (GSEA) of Gene Ontology (GO) biological process (BP) terms was carried out by the *fgsea* (v1.3) package in RStudio. Genes were ranked by their log2fold change in each DGE analysis. Adjusted p values < 0.05 were considered significant. Ontology gene sets were obtained from MsigDB [23]. To assess gene expression correlations between samples, pseudobulk analysis performed on aggregated gene expression per sample was determined using the “AggregateExpression” function in Seurat.

### Gene signature scores

M1 and M2 macrophage gene signature scores were created using the GSVA (v1.52.3) package for single-sample gene set enrichment analysis (ssGSEA). The list of external genes used to create this score is included in Supplementary Table S2. Statistical analysis was generated using the *ggpubr* wrapper package for *ggplot2* in RStudio.

### T cell receptor analysis

Single-cell TCR clonotypes were assembled and generated using the Cell Ranger VDJ function. Single-cell barcodes were used to correlate variable-diversity-joining (VDJ) gene segments with corresponding gene expression data, and these TCR clonotype data were analyzed using scRepertoire v1.8.0 for R. TCR clonotypes were defined based on shared CDR3α and CDR3β nucleotide sequences. A single sample from each individual patient and tissue site was individually evaluated for clonotype expansion (considered the “TCR pool”), which was grouped by the following criteria: “unique” for TCR clonotypes consisting of one clone; “rare” for clonotypes greater than one clone but less than 0.1% of the TCR pool; “small” for clones comprising 0.1% to less than 1% of the TCR pool; “medium” for clones comprising 1% to less than 2% of the TCR pool; “large” for clones representing 2% to less than 5% of the TCR pool; and “hyperexpanded” for clones consisting of greater than 5% of the TCR pool. CDR3 sequences were annotated for known viral sequences using the publicly available VDJdb database [24]. The diversity and overlap of TCR CDR3 sequences were analyzed using scRepertoire’s functions for rarefaction analysis, D50 diversity index, and the Jaccard index.

### Neoantigen-reactive T cell signature assessment

Gene signature scores were created with the AUCell R package and used to score tumor-infiltrating lymphocytes (TILs). Gene sets were derived from the following previously published studies: 40-gene version of Lowery NeoTCR4 [16], 243-gene version of NeoTCR8 [16], 98-gene version of Zheng ExRe CD4 [18], and 30-gene version of Meng TR30 [17]. We first filtered for TILs by identifying cells from the primary tumor that had a paired GEX and TCR data available. We determined the AUC score for each gene set and ranked cells according to their AUC score. We included the top 5% of ranked cells to nominate potentially reactive T cells, based on previously published methodologies [16]. Pseudotime trajectories of T cell differentiation were plotted using Monocle 3 v1.3.7.

### Statistical analysis

For cluster-based scRNA analysis, the “FindMarkers” function (using default values) within Seurat was used to identify differentially expressed genes between specific clusters, and the Wilcoxon rank-sum test was used to define significant values. Genes considered significant were included as DEGs. For pairwise comparison of immune cell populations between tissue sites and GSEA, Wilcoxon rank sum was used to determine statistical significance. For macrophage signature score analysis, the *ggpubr* plot wrapper package was used, and Wilcoxon rank sum was again employed to test for significance between tumor and liver macrophages. All analyses were performed in RStudio, and for all comparisons, an adjusted p-value threshold of < 0.05 was used to determine statistical significance.

## Results

### Construction of a multisite, immune cell atlas

To characterize multisite immunity at single-cell resolution, we performed paired scRNA-seq and scTCR-seq on immune cells derived from three chemotherapy-naïve patients with localized PDAC. The clinical characteristics and mutational status of these patients are shown in Supplementary Table S3. These three patients represent the spectrum of recurrence patterns following oncologic resection. Patient 027 experienced recurrence of disease with a liver metastasis seven months after resection, and patient 037 demonstrated early growth of an initially indeterminate liver lesion just five days after surgery. Each of these patients ultimately died of metastatic PDAC, at 25 and eight months from surgery, respectively. Patient 036 remains recurrence free and alive 27 months after diagnosis. Expansion of our patient panel will allow us to more comprehensively represent these recurrence patterns and more robustly test for correlations between immunological profile differences and clinical outcomes.

**Fig. 1:**
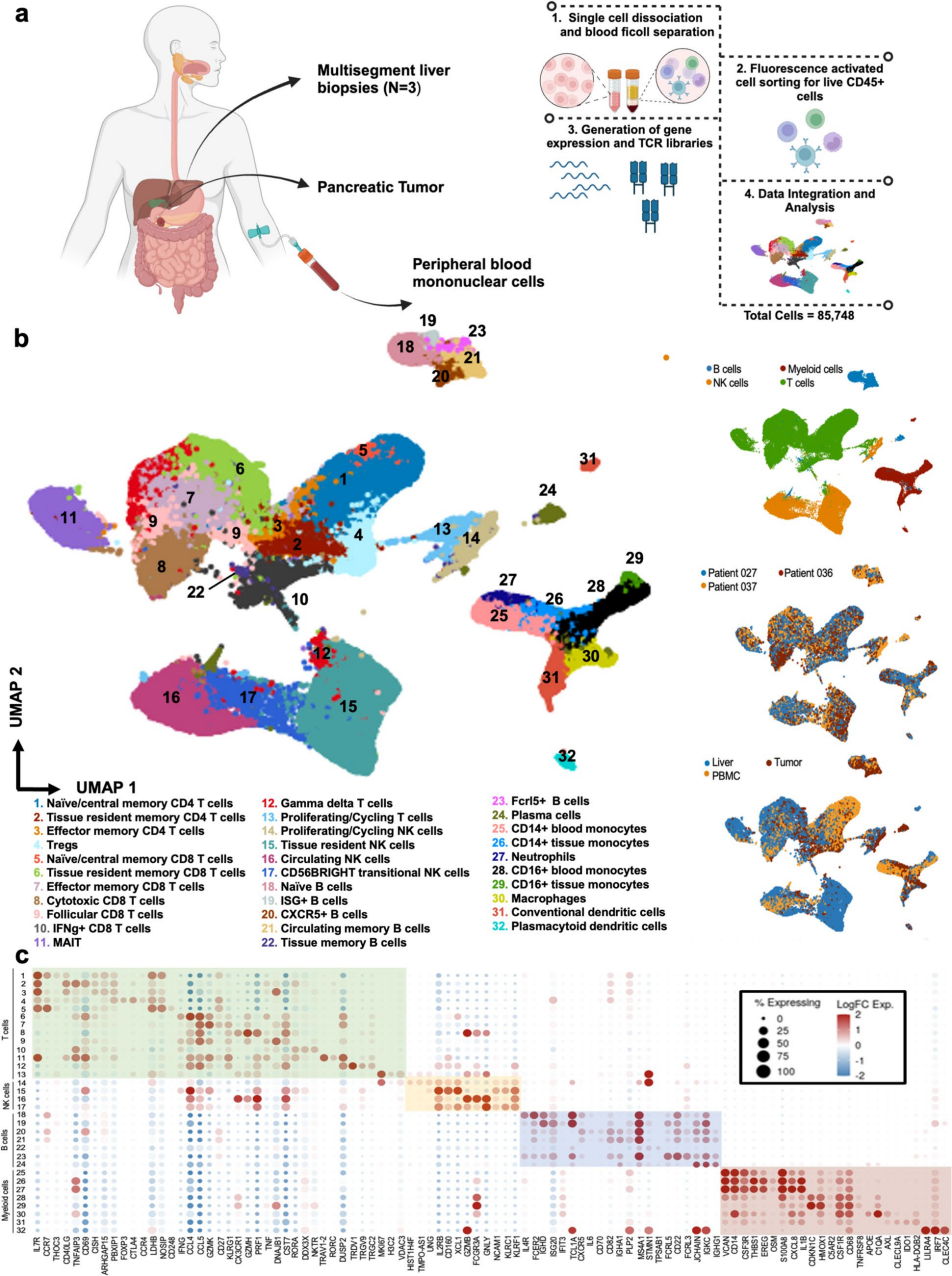
Single-cell RNAseq of immune populations in tumor, PBMC, and liver. **A** Schematic of the experimental workflow. Live CD45 + cells were isolated from multisegment liver biopsies, peripheral blood, and PDAC tumor of three patients with localized disease undergoing surgical resection. Paired gene expression and TCR libraries were then prepared, sequenced, and analyzed. Generated with BioRender. **B** Uniform manifold approximation and projection (UMAP) of 32 unique immune cell populations identified across all tissue sites. Cell types are depicted and grouped according to broad subclusters (T cells, NK cells, myeloid cells, and B cells) (top right insert), patient of origin (middle right insert), and tissue site of origin (bottom right insert). **C** Representative differentially expressed genes for each cell type are shown. Color gradient and dot size correlate with level of expression and percentage of cells expressing this gene, respectively

In total, 106,539 sorted CD45 + cells were captured (Fig. 1A), and after filtering for high-quality CD45 + cells, 85,748 cells were included for downstream analysis. Data were normalized and integrated to minimize batch effects and emphasize biological structure while conserving differences expected to be present in a multisite dataset. Gene expression-based annotation identified 32 unique cell populations across the three tissue types (Fig. 1B), revealing various subsets across each of the four major immune cell lineages (Fig. 1B, top insert). Cells labeled by patient demonstrated that all cell clusters were conserved across patients and were well integrated (Fig. 1B, middle insert). Cells labeled by the tissue site of origin revealed expected differences in cell-type composition (Fig. 1B, bottom insert). Figure 1C depicts representative differentially expressed genes (Supplementary Fig. S1 and Supplementary Table S1) used to guide annotation of each cluster.

One important component of our analysis was to evaluate the homogeneity of the immune composition across liver samples, specifically to determine the representative capacity of each 1 cm^3^ liver biopsy for the composite hepatic immune microenvironment. Leveraging our triplicate biopsies per patient, we correlated the immune cell composition, gene expression, and sharedness of TCR clonality for each liver sample from the same patient to assess concordance between biopsies (Supplementary Fig. S2A–E). For immune cell composition we show both the correlation of cell-type proportions between liver replicates and across patients and percent of each sample for each annotated cell type (Supplementary Fig. 2SA–C). For gene expression, we performed pseudobulk analysis by averaging gene expression for each sample and show the correlation between samples (Supplementary Fig S2D). TCR clonality sharedness is demonstrated using the Jaccard index, showing significant overlap of TCR clones in the liver replicates (Supplementary Fig S2E). Indeed, conservation was demonstrated across the multisegment specimens, confirming that a single 1 cm^3^ biopsy can provide adequate representation of the larger hepatic immune microenvironment for an individual patient.

### Site-specific immune profiling

**Fig. 2:**
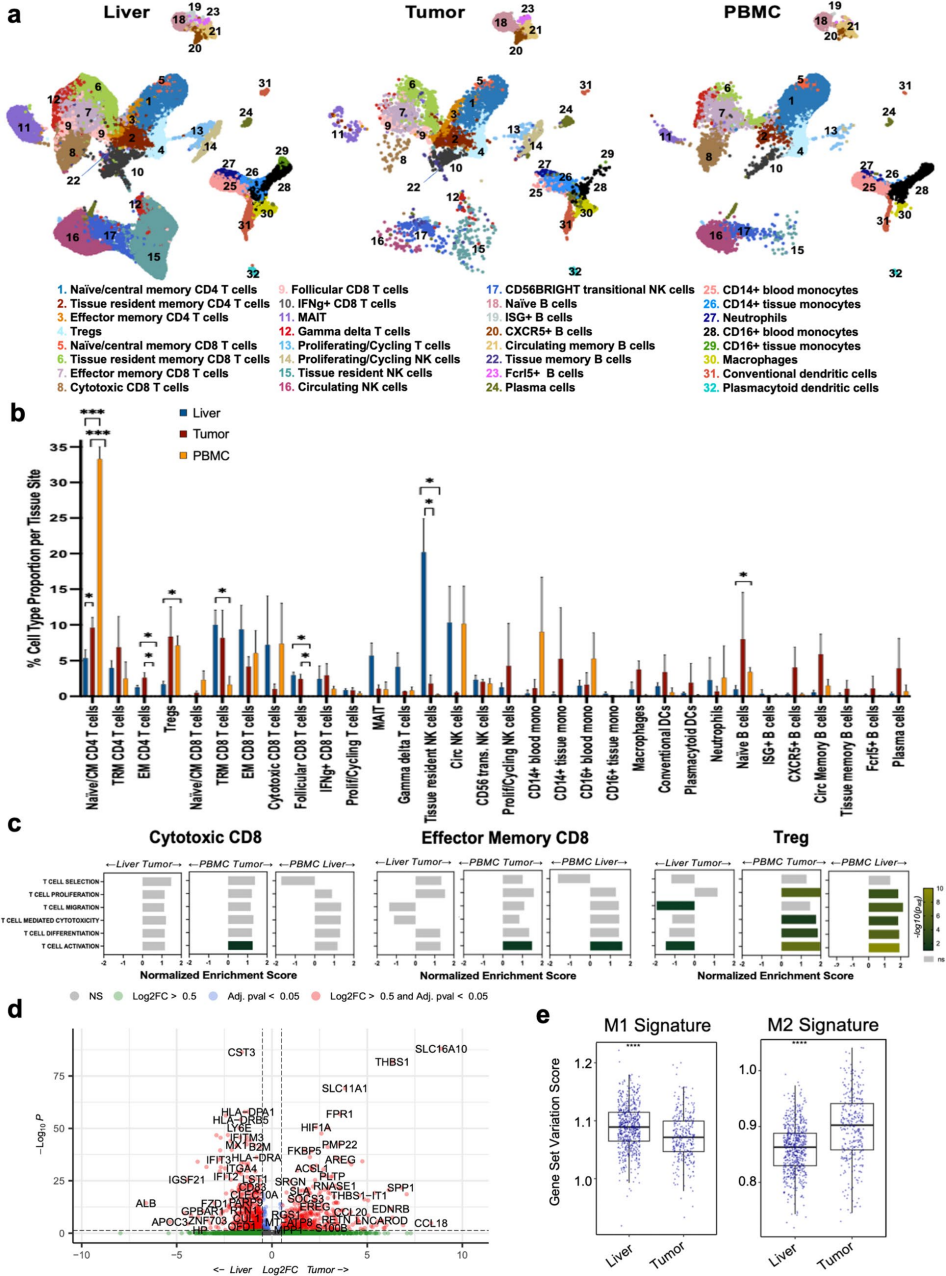
Composition and function of cells across tissue sites. **A** UMAP of the identified 32 immune cell types stratified by tissue site of origin. **B** Barplots depicting the mean cell-type abundance for each tissue site. Wilcoxon rank sum was used for statistical analysis. Error bars represent the standard deviation across the three patients. *p ≤ 0.05, ***p ≤ 0.001 **C** Three-way comparison of gene set enrichment analysis (GSEA) for GO. BP immune cell pathways among cytotoxic CD8, effector memory CD8, and Treg cells within the different tissue sites. Bars are colored according to p-value. **D** Volcano plot illustrating differentially expressed genes between macrophages isolated from the tumor (right) versus the liver (left). Dotted lines indicate p_value_ < 0.05 and |log_2_(FC)|> 0.5. **E** Boxplots representing M1 and M2 gene signature scores for liver and tumor macrophages, using gene set variation analysis (GSVA)

We next evaluated differences in the composition and transcriptional activity of immune populations within each tissue site. One goal of this pipeline is to demonstrate the feasibility of deep immune profiling of the liver and tumor by sorting for immune populations prior to single-cell analysis. First, we calculated the proportion of each cell type based on tissue site. While most cell types were represented across all sites, the composition expectedly varied by location, and certain resident cell types were only found within their respective tissues, as described below (Fig. 2A, B). Notably, interpatient variation was relatively limited across all cell types and tissue sites (Supplementary Fig. S2A, C, D). The greatest interpatient immune heterogeneity was found within the tumor microenvironment (Supplementary Fig. S2A, D). This was consistent with differences previously seen in tumor studies of PDAC patients, some of which have been shown to ultimately impact survival [25–29].

Within the PBMCs, the most abundant cell type was naïve/central memory CD4 T cells (32.9%), followed by CD14 + blood monocytes (12.0%), consistent with the need for broad immune surveillance. As expected, low levels of resident cell types were found in the blood. The liver was most enriched for NK cells, with tissue resident (20.4%) and circulating NK cells (10.2%) being the most abundant cell types. There was also enrichment for differentiated CD8 + T cell populations in the liver, with tissue resident memory (9.9%), effector memory (9.3%), and cytotoxic (7.4%) CD8 T cells as the next most abundant cell types. Within the tumor, naïve/central memory CD4 + T cells (9.4%), naïve B cells (9.1%), Tregs (7.8%), and tissue resident memory CD8 + T cells (7.1%) were the most abundant cell types, demonstrating a broad immune complement. The tumor also showed a larger proportion of B cells relative to the other sites, consistent with other reports of tumor-infiltrating B cells [29] (Fig. 2A, B).

To explore differences in immune phenotypes across the various sites, we performed three-way gene set enrichment analyses, focusing on terminally differentiated CD8 + T cell populations and Tregs, both of which are critical for antitumor immunity. Within both the tumor and the liver relative to the blood, we saw an enrichment of genes associated with T cell activation and function (Fig. 2C, Supplementary Table S4). In contrast, we identified significant enrichment in the proliferation, activation, and differentiation pathways of Tregs within the tumor and liver relative to those in the blood (Fig. 2C). This is consistent with the immunosuppressive microenvironment present within the tumor and the immunoregulatory state of the liver [8, 9, 25, 26, 28].

Compared to the PBMCs, liver cytotoxic and effector memory CD8 T cells overexpressed key marker genes of T cell activation such as *JUNB, CD69, CXCR6,* and *ICOS (CD278)* (Supplementary Fig. S3A). Additionally, they overexpressed common markers of T cell function and chemoattraction such as *CCL4, TNF,* and *IFNG. JUNB, JUND,* and *FOS,* which are among the most overexpressed genes in these liver cell types, are transcription factors belonging to the activated protein (AP-1) family and are critical for effector CD8 T cell differentiation and cytokine production [30]. *DUSP1 (MKP-1)*, another highly expressed gene in liver terminally differentiated T cells relative to the PBMCs, is a necessary mediator of antigen-specific T cell activation [31] (Supplementary Fig. S3A, B, Supplementary Table S5). Overall, liver effector CD8 T cells exhibited an activated cytolytic profile.

Specific to tumor cytotoxic T cells, *DUSP4* was overexpressed relative to the liver and PBMCs. *DUSP4* is implicated in the development of T cell exhaustion [32], and we also identified overexpression of several other classically defined exhaustion marker genes such as *ENTPD1*, *IL2RA,* and *CXCR6*, consistent with an immune-exhausted tumor microenvironment (Supplementary Fig. S3A, Supplementary Table S5). Tumor effector memory CD8 T cells demonstrated overexpression of *CCR7, IL7R,* and *CXCR4,* which are associated with a naïve-like T cell state and inversely associated with T cell polarization and effector T cell function [33–35], as well as *TXNIP,* which negatively regulates T cell proliferation [36] (Supplementary Fig. S3B, Supplementary Table S5). Altogether, terminally differentiated liver CD8 T cells appeared more primed for T cell immune activity, while tumor CD8 T cells simultaneously exhibited markers of activation, counterbalanced by T cell exhaustion and negative regulation of effector function.

We next explored immune suppression, focusing on Tregs, which displayed the greatest intersite differences in GSEA results (Fig. 2B, C). Notably, tumor and liver Tregs showed greater enrichment of gene sets related to activation, recruitment, and function relative to blood Tregs (Fig. 2C). This is consistent with the known immunosuppressive microenvironment of primary PDAC [25, 26, 37] and normal liver [8, 9] and may partially explain the liver tropism for metastases in PDAC*.* Liver Tregs, although comprising a minority of the hepatic immune composition, demonstrated higher activity and migration scores than even those within the tumor. DGE analysis revealed that liver Tregs, compared to tumor and PBMC Tregs, had higher expression of key genes linked to Treg recruitment and immune suppression (e.g., *CXCR3, CCL3, CCL4,* and *CCL5*) [38–41] (Supplementary Fig. S3C, Supplementary Table S5). Tumor Tregs, compared to the liver and blood, showed upregulation of markers of Treg activation and cancer-associated immune suppression, such as *IL2RA, IL1R2, FOXP3, TIGIT, CTLA4, ENTPD1, CXCL13, CXCR4,* and *RGS1* (Supplementary Fig. S3C, Supplementary Table S5). Altogether, this depicted Treg-driven immunosuppression within the tumor and the premetastatic liver that is consistent with PDAC being considered immunologically “cold” [25, 26, 28, 37] and highlights a potential cause for hepatic recurrence patterns.

Lastly, as tissue macrophages are also known to contribute to PDAC immunosuppression, we evaluated functional differences between liver and tumor macrophages. Tumor macrophages demonstrated significant upregulation of genes associated with polarization to the tumorigenic and immunosuppressive M2 phenotype (e.g., *APOE, CD163, WNT5A,* and *THBS1)* (Fig. 2D, Supplementary Table S6). There was also upregulation of myeloid-derived suppressor cell (MDSC) marker genes (*S100A8, S100A9, VEGFA,* and *CD84)* as well as of *SLC16A10,* a marker of tumor-associated macrophages (TAM) (Fig. 2D, Supplementary Table S6). The tumor macrophages displayed prominent upregulation of *HIF1A*, a gene that when detected on TAMs has been implicated in cancer progression, T cell suppression, and metastasis [42, 43]. Notably, macrophages from the tumor also had a significant upregulation of *SPP1*, an M2 marker recently reported to be a strong prognostic indicator of poor outcomes [44] (Fig. 2D, Supplementary Table S6). Indeed, tumor macrophages scored higher for an overall M2 signature [45] compared to the liver (Fig. 2E). One of the highest expressed genes among liver macrophages, *CST3*, is associated with immune inactivation [46]. Liver macrophages also had high expression of *APOC3*, a known activator of the NLRP3 inflammasome. NLRP3 inflammasome activation in macrophages has been shown to drive colorectal cancer metastasis to the liver [47]. Taken together these data show a profile of immunosuppressive macrophages with distinct phenotypes of suppression in the tumor and liver, a finding requiring additional follow-up with larger sample sizes and clinical outcomes to provide the necessary context for interpretation.

### Tracking clonal expansion across blood and tissue sites

**Fig. 3:**
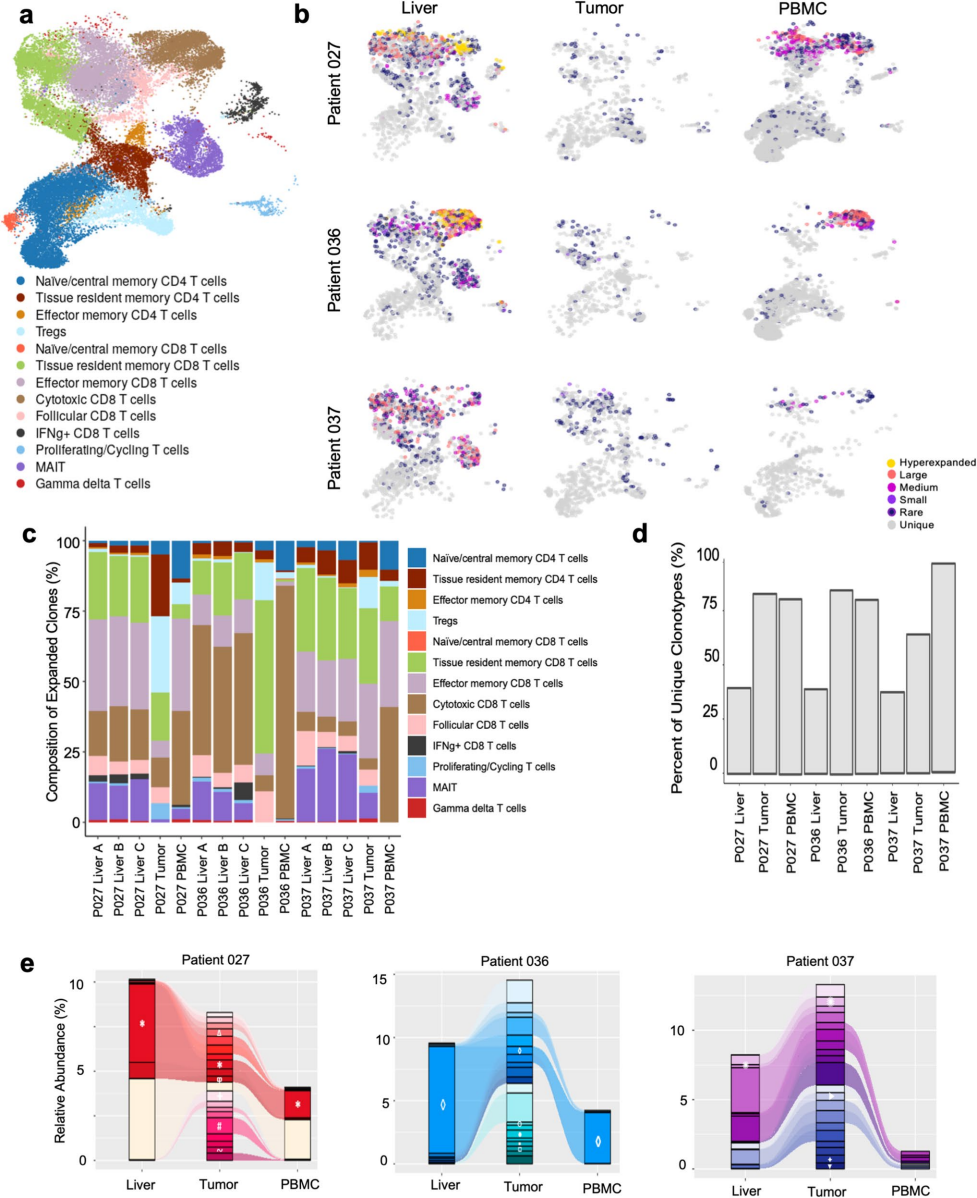
T cell receptor repertoire analysis. **A** Filtered UMAP to include only T cells with paired gene expression and T cell receptor (TCR) sequencing data. **B** UMAP of TCR clonotype expansion subset by patient and tissue site of origin. Clonal expansion was defined as the following: “unique” for TCR sequences of only one clone; “rare” for one clone ≤ 0.1%; “small” for 0.1% ≤ 1%; “medium” for 1% ≤ 2%; “large” for 2% ≤ 5%; and “hyperexpanded” for > 5% of the TCR pool. **C** The cell-type composition of expanded clones from each sample. **D** Percent of unique clones in the TCR pool from each sample. **E** Top 20 expanded TCR clones within each tumor sample tracked to the liver and PBMC dataset for each patient. Clones with CDR3 sequences annotated to known viral antigens are depicted by white symbols

Clonal expansion has been shown to be a marker of a robust antitumoral T cell response in PDAC [48–51], and thus, we next characterized T cell clonal expansion across tissue sites. Expanded clones were predominantly comprised of CD8 T cells, with terminally differentiated CD8 T cells accounting for > 50% of expanded clones in 14 of 15 samples (Fig. 3B). These observations are consistent with previous experimental studies demonstrating that CD4 T cells exhibit limited clonal expansion and proliferation relative to CD8 T cells [52, 53].

Within the liver, terminally differentiated CD8 T cells comprised the majority of expanded clones, yet there was considerable variability between patients regarding the expansion patterns of specific cell types. In patient 027, liver-expanded clones were relatively evenly comprised of cytotoxic, effector memory, and tissue resident CD8 T cells, while the dominant expanded cell types were cytotoxic CD8 T cells in patient 036 and tissue resident memory CD8 T cells in patient 037. There were only modest differences in clonal expansion patterns between triplicate liver samples from each patient, consistent with liver homogeneity between segments (Fig. 3B, Supplementary Fig. S2E). While CD8 T cells were the predominant expanded clones within the tumor, they were less expanded relative to the other tissue sites. Notably, intratumoral clonally expanded Tregs were observed in all three patients, while these were rarely observed in the liver or peripheral blood (Fig. 3B).

PBMCs had the greatest proportion of unique clonotypes (Fig. 3C), with the majority of cells representing unique singletons. This greater clonal diversity relative to the liver and tumor was confirmed using both the D50 diversity index and rarefaction analysis (Supplementary Fig. S4A-B). Like the other sites, the majority of blood expanded clones were mature CD8 T cells, and relative to these other sites, more naïve/central memory CD4 T cells were observed in the blood, while populations such as follicular CD8 T cells were essentially absent. Altogether, these findings suggest important patterns in clonal expansion, trafficking, and retention of T cell clones based on tissue location, with additional patient-level diversity, the importance of which requires follow-up in larger studies.

We next evaluated whether tumor-expanded T cell clones were also detected and clonally expanded within other tissue compartments, which we hypothesized might represent a premetastatic antitumoral immune response. The top twenty most expanded tumor clones were identified for each patient (Fig. 3D) and tracked across the sites. Because it has also been shown that a subset of clonally expanded T cells within the tumor microenvironment are bystander cells without specificity toward tumor antigens [54, 55], the CDR3 sequences from our dataset were checked against previously annotated sequences [56] of known viral antigens (Supplementary Table S7), acknowledging the inability to confirm viral mapping without knowledge of each patient’s HLA type.

The pattern of sharedness between T cell clones in the tumor and the liver/blood varied by patient. In patient 027, eight and 12 of the 20 tumor-expanded TCRs were also detected within the liver and blood, respectively. Within the liver, the second most expanded of these shared clones, accounting for 4.5% of the liver TCR pool, was linked to a known viral antigen. This clone was also expanded in the blood, comprising 1.5% of the blood TCR pool, likely representing a previous viral response. The remaining shared clones within the liver did not map to any known antigen, including another prominently expanded clone (comprising 4.7% of the TCR pool). In patient 036, 11 and four of the 20 tumor-expanded clones were detectable in the liver and blood, respectively. Of these shared clones, the largest expanded clone in both the liver and blood (comprising 8.3% of the TCR pool of the liver and 4.0% of the blood) had a known CDR3 sequence annotated to cytomegalovirus [24], again likely indicating a response to a previous infection. The remaining shared clones within the liver, although not linked to a viral antigen, were expanded to less than 0.5% of the TCR pool each. In patient 037, 12 and seven of the 20 tumor-expanded clones were also present in the liver and blood, respectively. Only one clone within the liver, expanded to just 0.39% of the liver TCR pool, mapped back to a known viral antigen. The remainder of the shared clones, including clones expanded up to 2.7% of the TCR pool, could not be annotated to known viral antigens (Fig. 3D). Overall, while clonal expansion may indicate signs of antitumoral immune activity, our data corroborate substantial clonal activation toward viral antigens, thus demonstrating the presence of bystander T cells and emphasizing that clonal expansion alone is not enough to identify tumor reactivity.

### Leveraging paired scRNA-seq and scTCR-seq to predict tumor reactivity

**Fig. 4:**
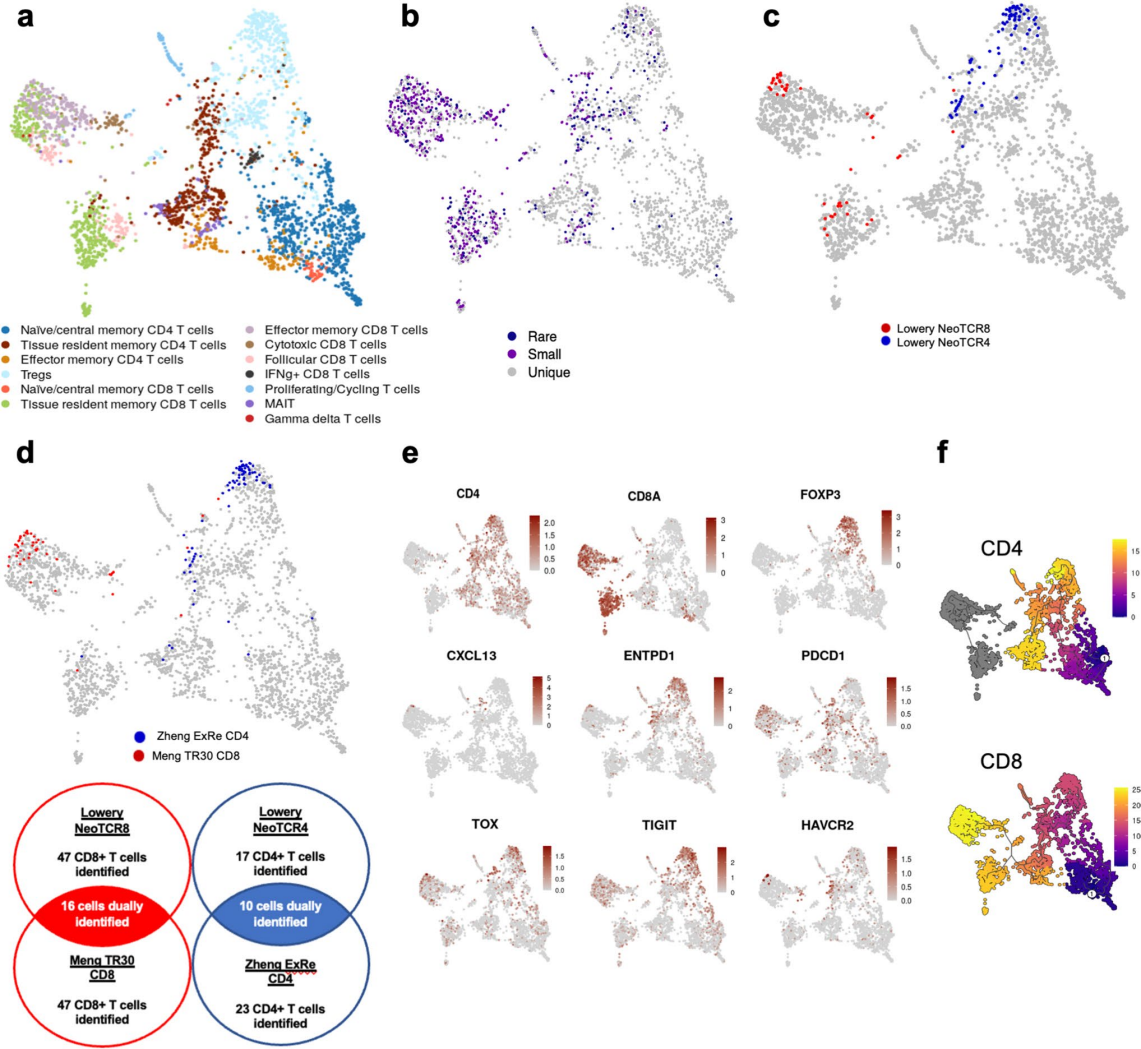
Neoantigen reactivity scores of tumor T cells. Filtered UMAP to include only T cells from the tumor colored by **A** cell types or **B** clonal expansion. Clonal expansion was defined as the following: “unique” for TCR sequences of only one clone; “rare” for one clone ≤ 0.1%; “small” for 0.1% ≤ 1%. **C** Cells that score in the 95th percentile of NeoTCR4 and NeoTCR8 signatures are highlighted. **D** Cells that score in the 95th percentile of Zheng ExRe tumor-reactive CD4 and Meng TR30 tumor-reactive CD8 signature scores are highlighted. Venn diagram summarizing the number of cells that scored for each gene signature with dually scored “consensus” cells shown in the overlap. **E** Feature plots visualizing gene expression of key marker genes associated with the tumor reactivity signatures. **F** Pseudotime trajectory analysis of CD4 and CD8 T cell differentiation. Color gradient represents degree of cell differentiation, from least (purple) to most (yellow) differentiated subclusters

Several groups have recently shown that tumor-reactive T cells share unique transcriptomic features associated with activation, dysfunction, and differentiation [16, 18, 55, 57], and that paired scRNA-seq and scTCR-seq can be leveraged to predict TCRs with specificity to tumor antigens [16–18]. Thus, we sought to apply previously published, validated gene expression signatures [16–18] to predict tumor-reactive CD4 and CD8 T lymphocytes. Restricting the analyses to only tumor T cells with paired scRNA-seq and scTCR-seq data, TILs were re-clustered (Fig. 4A). Consistent with our prior analysis, clonal expansion was more commonly observed in CD8 T cells than in CD4 T cells (Fig. 4B).

We then applied three separate gene signatures to predict tumor reactivity: Lowery (CD4 and CD8 T cell prediction) [16], Meng (CD8 T cell prediction) [17], and Zheng (CD4 T cell prediction) [18]. Tregs were filtered out of analyses, as it is known that this population shares a similar transcriptional phenotype without being tumor reactive [58–60]. Other excluded T cells from this analysis were MAIT cells, which have semi-invariant TCRs and are identified by TRAV1-2^+^ TCRα chain expression, gamma delta (γδ) T cells, which lack αβ chains, and proliferating/cycling T cells, which were transcriptionally limited to cell cycle genes. Across prediction models, the top 5% of potentially tumor-reactive CD8 and CD4 T cells occupied clear clusters of predominantly tissue resident/effector memory CD8 T cells and tissue resident memory CD4 T cells, respectively (Fig. 4C, D, Supplementary Fig. S5A, B). Notably, 16 (34%) CD8 T cells dually scored for both Lowery and Meng predictive CD8 signatures, while 10 CD4 T cells were dually identified by both Lowery and Zheng predictive CD4 signatures (58.8% of the Lowery NeoTCR4 and 43.5% of the Zheng ExRe CD4) (Fig. 4D), providing a high-priority consensus list of TCR sequences with potential tumor reactivity.

These nominated clusters upregulated key marker genes of activation, dysfunction, and differentiation that have been used to identify tumor specificity [16–18], such as *CXCL13, TIGIT, PDCD1 (PD1), ENTPD1 (CD39), TOX, HAVCR2 (TIM-3),* and *LAG3* (Fig. 4E). Pseudotime trajectory analysis of T cell differentiation further identified these clusters as the most differentiated T cell populations (Fig. 4F), consistent with previous studies [16]. While these findings warrant further experimental validation to test the reactivity of nominated TCRs and identify the recognized antigen, these data demonstrate the potential power of single-cell approaches to overcome the challenge of distinguishing tumor-reactive from bystander TILs and are currently being incorporated into our workflow to develop PDAC cell-based therapies.

Finally, we tracked the consensus CD4 and CD8 T cell clones to determine their presence in the premetastatic sites. We identified a small panel of consensus CD8 T cell clones with varying degrees of clonal expansion that were detectable within the liver samples of each of the three patients (Supplementary Figure S5C). This demonstrates the feasibility of incorporating these signatures into clinical immune monitoring as an additional biomarker. Follow-up studies with larger patient cohorts will experimentally validate these predictive gene signatures within PDAC and investigate whether the presence of these nominated tumor-reactive clones within the premetastatic liver is associated with clinical outcomes and recurrence patterns.

## Discussion

While there is a growing body of work characterizing PDAC intratumoral immunity, there are limited data studying premetastatic systemic immunity, and hepatic immunity remains largely unexplored. Here, we designed a pilot study to generate a multisite platform for immune profiling of premetastatic PDAC. Within our pilot program, we identified cell populations across tissue sites that were reproducible across patients. Consistent with prior studies, we demonstrated that many clonally expanded intratumoral T cells mapped back to TCRs known to recognize viral antigens, and thus we employed predictive tools for the identification of candidate tumor-reactive T cells based on published, validated transcriptional signatures. This provides a platform for in-depth exploration of tissue-specific immunity in PDAC which has already been implemented within existing clinical trials and protocols, including one testing liver-directed therapy to reduce hepatic recurrence [19] and another testing the use of T cell therapy to target early recurrence [61, 62].

We sought to demonstrate the feasibility of this platform and highlight ways that it could be more widely applied within larger studies. One important technical question was whether a single 1 cm^3^ liver biopsy was representative of the larger hepatic immune compartment. Indeed, we found that the liver immune milieu is preserved across multisegment biopsies, and thus, ongoing and future efforts have utilized a single liver biopsy for single-cell applications, which improves throughput and reduces cost restraints inherent to this type of work, allowing for future use of this pipeline to generate data from a broader set of patients.

One recently published study provided the first depicture of how features from the premetastatic liver might be associated with outcomes and recurrence in human PDAC patients [63]. Methods applied in this study included bulk RNA sequencing, histologic analyses, and metabolomic profiling on archived liver samples. Their findings demonstrated that premetastatic livers in PDAC were marked by increased inflammation and changes in immune composition. They reported that, among cancer patients, increased inflammatory activity was considered a signature of resistance to liver metastasis. scRNA-seq of hepatic immune cells was performed to explore differences in broad cell types between premetastatic PDAC and control non-cancer livers, although granular data on immune cell subsets were not available. Here, we have gone a step further to show that paired scRNA-seq and scTCR-seq libraries can identify very granular differences in T cell clonal activation and expansion at the patient- and tissue-specific level. While our current study was not powered to test how trends in immunity are associated with clinical outcomes, this platform provides a pipeline to build on the work of Bojmar and others. Indeed, within our small study we see three distinct recurrence patterns that may associate with immunological patterns observed here. Ongoing studies are focused on performing deeper analyses of anti-tumor immunity within the premetastatic liver in a larger cohort that is powered to define how it fits within the context of intratumoral and systemic immunity.

There are several limitations to this study. First, our small sample size was intentionally designed to generate a pipeline that can be used in larger ongoing and future studies; however, this limits our ability to meaningfully correlate the presented findings with clinical patient outcomes. Nonetheless, we anticipate that this will be possible as we expand this pipeline to a currently accruing clinical trial from our group with a substantially expanded patient panel, which will capture both clinical factors (e.g., prior chemotherapy, biliary drainage) and patient outcomes (e.g., short- vs. long-term progression-free survival). Second, while we applied previously validated gene signatures to predict putative tumor-reactive clones, this present analysis was not designed to experimentally validate tumor reactivity of the nominated TCRs. Instead, via an ongoing collaboration to generate PDAC cell therapy [61, 62], we will have the necessary resources to apply this platform with the added infrastructure to perform whole-exome sequencing, identify tumor neoantigens, and archive lymphocytes and antigen-presenting cells to functionally validate nominated tumor-reactive TCRs. In addition, it is important to note that the signatures developed in Lowery et al. relied on metastatic samples, while both Meng et al. and Zheng et al. utilized primary tumors in their development [16–18]. Understanding the differences in primary and metastatic anti-tumor immunity is a critical open area of investigation and worth considering when interpreting the application of these signatures more broadly. Lastly, single-cell sequencing does not provide spatial context about the interaction of immune cells within the liver. To add this critical element, we are employing spatial profiling techniques in combination with our single-cell pipeline to evaluate the organization of immune cell neighborhoods across the hepatic landscape.

In conclusion, our study describes a platform for in-depth analysis of tissue-specific immunity in the context of premetastatic PDAC. This lays the critical groundwork to expand these efforts to a larger panel of patients with localized PDAC and investigate how differences in intratumoral, systemic, and hepatic immunity shape metastatic progression. Our hope is that a better understanding of these features will permit the development of novel approaches to prevent or delay recurrence in this otherwise recalcitrant disease. As we continue to develop biomarkers to characterize and predict the response of patients to therapies, this pipeline can be applied to build a temporal immune profile regardless of tumor type or stage.

## Supplementary Information

Below is the link to the electronic supplementary material.

Supplementary file1 (DOCX 15 KB)

Supplementary file2 (TIFF 17372 KB)

Supplementary file3 (TIFF 17372 KB)

Supplementary file4 (TIFF 17372 KB)

Supplementary file5 (TIFF 17372 KB)

Supplementary file6 (TIFF 17372 KB)

Supplementary file7 (XLSX 5425 KB)

Supplementary file8 (XLSX 10 KB)

Supplementary file9 (DOCX 19 KB)

Supplementary file10 (XLSX 33 KB)

Supplementary file11 (XLSX 5459 KB)

Supplementary file12 (XLSX 647 KB)

Supplementary file13 (XLSX 40 KB)

## Data Availability

All sequencing data generated by this study and supporting the findings of this study have been deposited in a publicly available open source database accessible at the following location: https://github.com/afletch00/Kanu_Liver_scRNA.git.
