# Supplementary material for: A platform for multisite immune profiling of premetastatic pancreatic cancer at single-cell resolution
Source: Cancer Immunol Immunother. 2025 Aug 23;74(9):291. doi: 10.1007/s00262-025-04146-5 (PMC12374926; doi:10.1007/s00262-025-04146-5)
Supplement: Supplementary file 1 — Supplementary file1 (DOCX 15 KB) [file 262_2025_4146_MOESM1_ESM.docx]

**Supplementary Figure Legends:**

**Supplementary Figure S1. Processing and QC of scrNAseq** (A) Cells were filtered for quality control based on detected gene features (minimum and maximum cutoff values of 300 and 6000 respectively), total RNA counts per cell (minimum and maximum cutoff values of 500 and 25,000), and detected mitochondrial genes (with a maximum detection cutoff of 15%). (B) Cells obtained pre- and post- QC filtration for each sample are listed in a table. Representative differentially expressed genes for (C) NK cells, (D) myeloid cells, (E) B cells, and (F) T cells used to guide subcluster annotations are shown. Color gradient and dot size correlate with level of expression and percentage of cells expressing this gene, respectively.

**Supplementary Figure S2. Homogeneity across replicate liver biopsies** (A) The immune cell composition of each sample is plotted in a correlation matrix to assess concordance across samples. Color gradient correlates with higher concordance. (B) The correlation values seen in (A) for each pairwise comparison between liver vs liver and liver vs non-liver samples, based on cell type proportion, was plotted, and the distribution of correlation values was visualized in boxplot form. (C) The percentage of each immune cell type within the liver samples of each patient are visualized in a heatmap. Color gradient correlates with higher cell type enrichment. (D) The average expression per gene of each sample, using pseudobulk analysis, is plotted in a correlation matrix, to assess concordance across samples. Color gradient correlates with higher concordance. (E) Jaccard index correlation of expanded clones across samples for each patient. TCRs were filtered to exclude singleton clones. Color gradient correlates with higher concordance.

**Supplementary Figure S3. Volcano plots for DEGs.** Three-way comparisons of differentially expressed genes of (A) effector memory CD8 T cells, (B) cytotoxic CD8 T cells, and (C) Tregs between the tumor versus liver, tumor versus PBMC, and liver versus PBMC. Dotted lines indicate p_value_ < 0.05 and |log_2_(FC)| > 0.5.

**Supplementary Figure S4. TCR clonality analysis** (A) D50 diversity index estimating the clonal diversity for each sample. Barplot represents the number of unique clones occupying 50% of the total TCR repertoire for each sample. (B) Rarefaction analysis estimating the diversity within each sample, as measured by the estimated number of unique TCR clones within each sample as a function of the sample size.

**Supplementary Figure S5. Predicted neoTCRs for each patient.** Filtered UMAP to include only T cells from the tumor stratified by patient. Cells that score in the 95th percentile of Lowry NeoTCR4 and NeoTCR8 (A) or Zheng ExRe tumor reactive CD4/ Meng TR30 tumor reactive CD8 (B) signature scores are highlighted. (C) Bubble plot depicting consensus scored TCRs from the tumor of each patient tracked across liver and PBMC. Color and size correlate to tissue site location and magnitude of TCR clonal expansion, respectively.
