# Supplementary figures and images for: A platform for multisite immune profiling of premetastatic pancreatic cancer at single-cell resolution
Source: Cancer Immunol Immunother. 2025 Aug 23;74(9):291. doi: 10.1007/s00262-025-04146-5 (PMC12374926; doi:10.1007/s00262-025-04146-5)

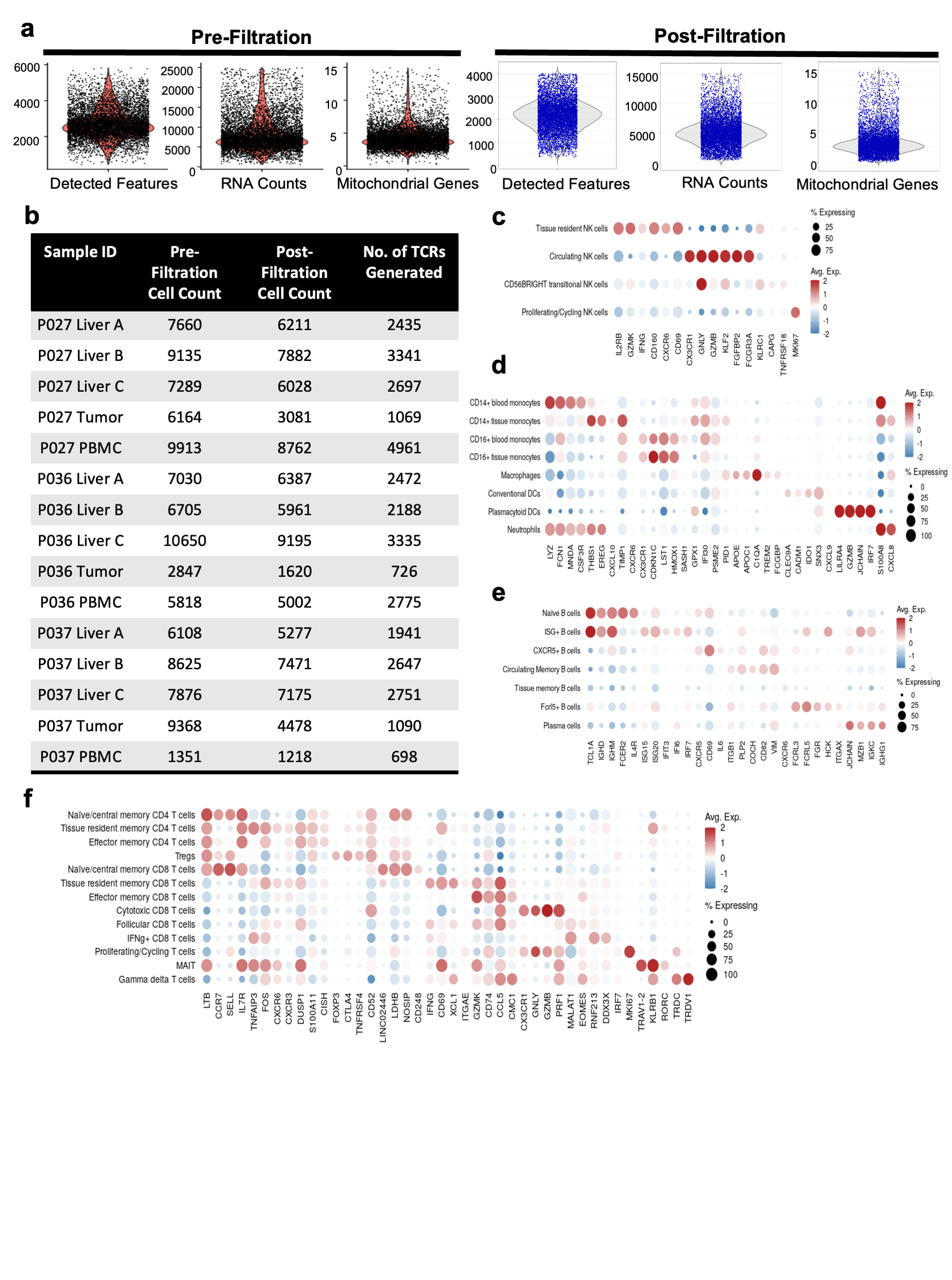

Supplement: Supplementary file 2 — Supplementary file2 (TIFF 17372 KB) [file 262_2025_4146_MOESM2_ESM.tiff]

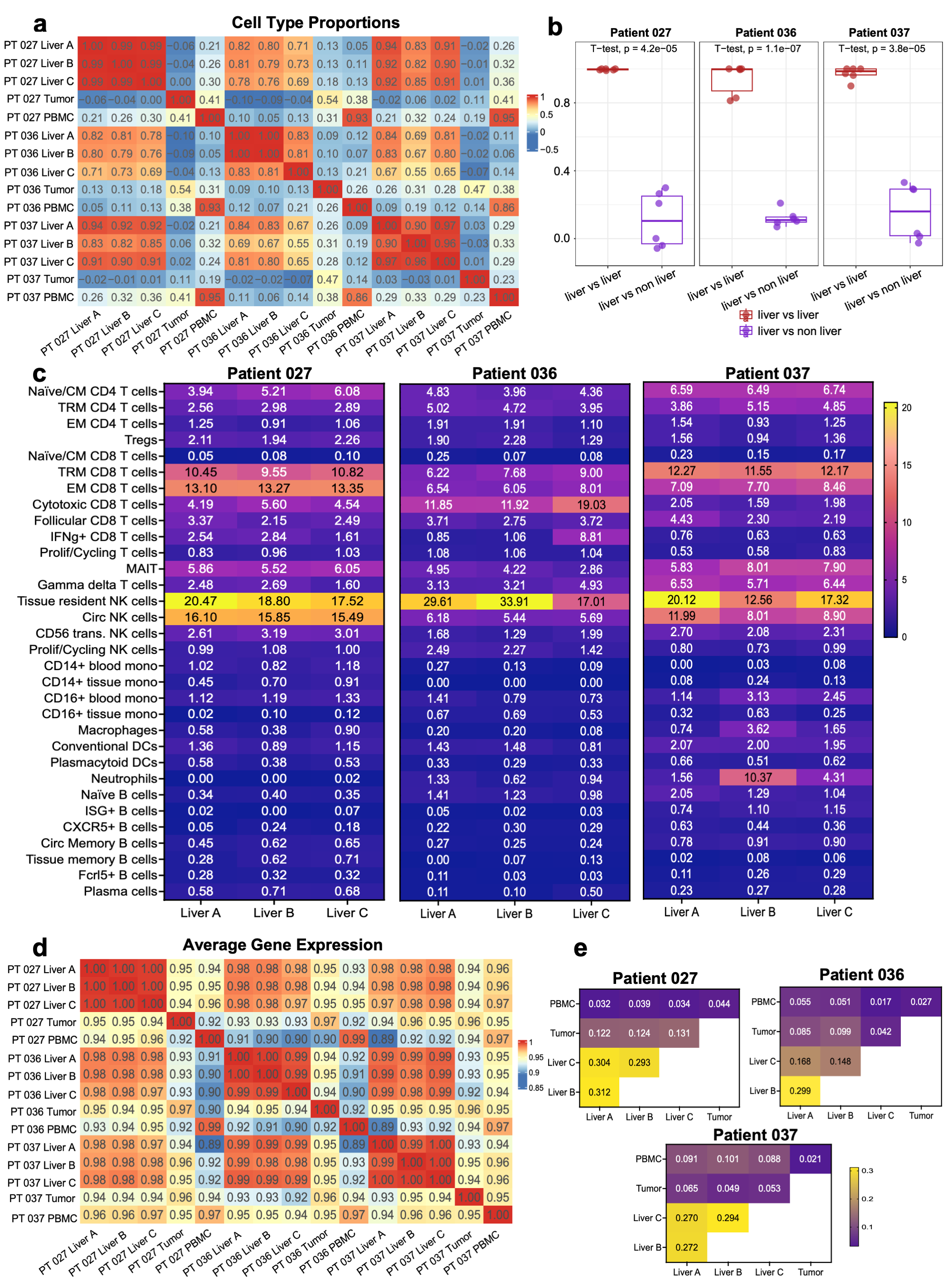

Supplement: Supplementary file 3 — Supplementary file3 (TIFF 17372 KB) [file 262_2025_4146_MOESM3_ESM.tiff]

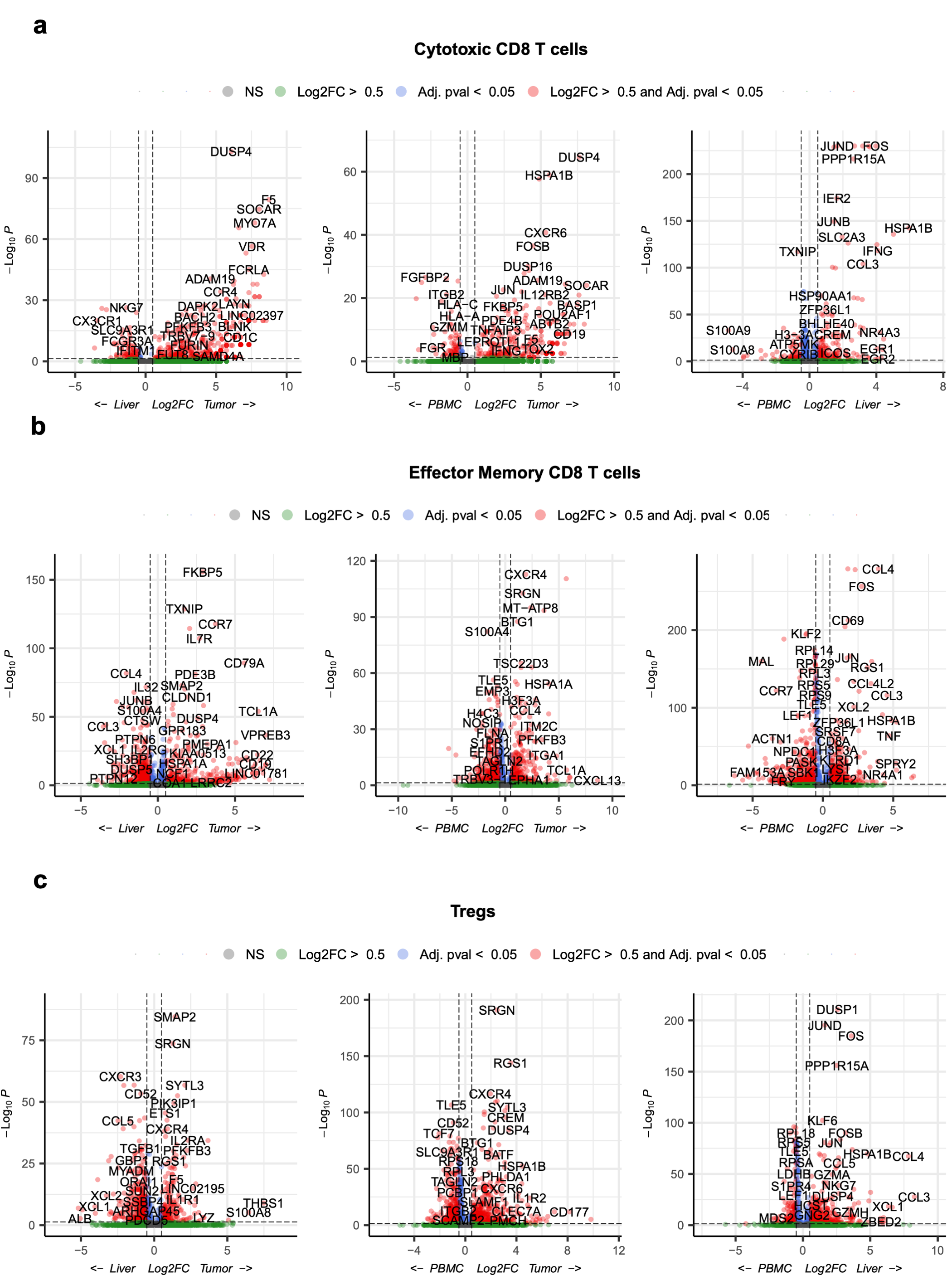

Supplement: Supplementary file 4 — Supplementary file4 (TIFF 17372 KB) [file 262_2025_4146_MOESM4_ESM.tiff]

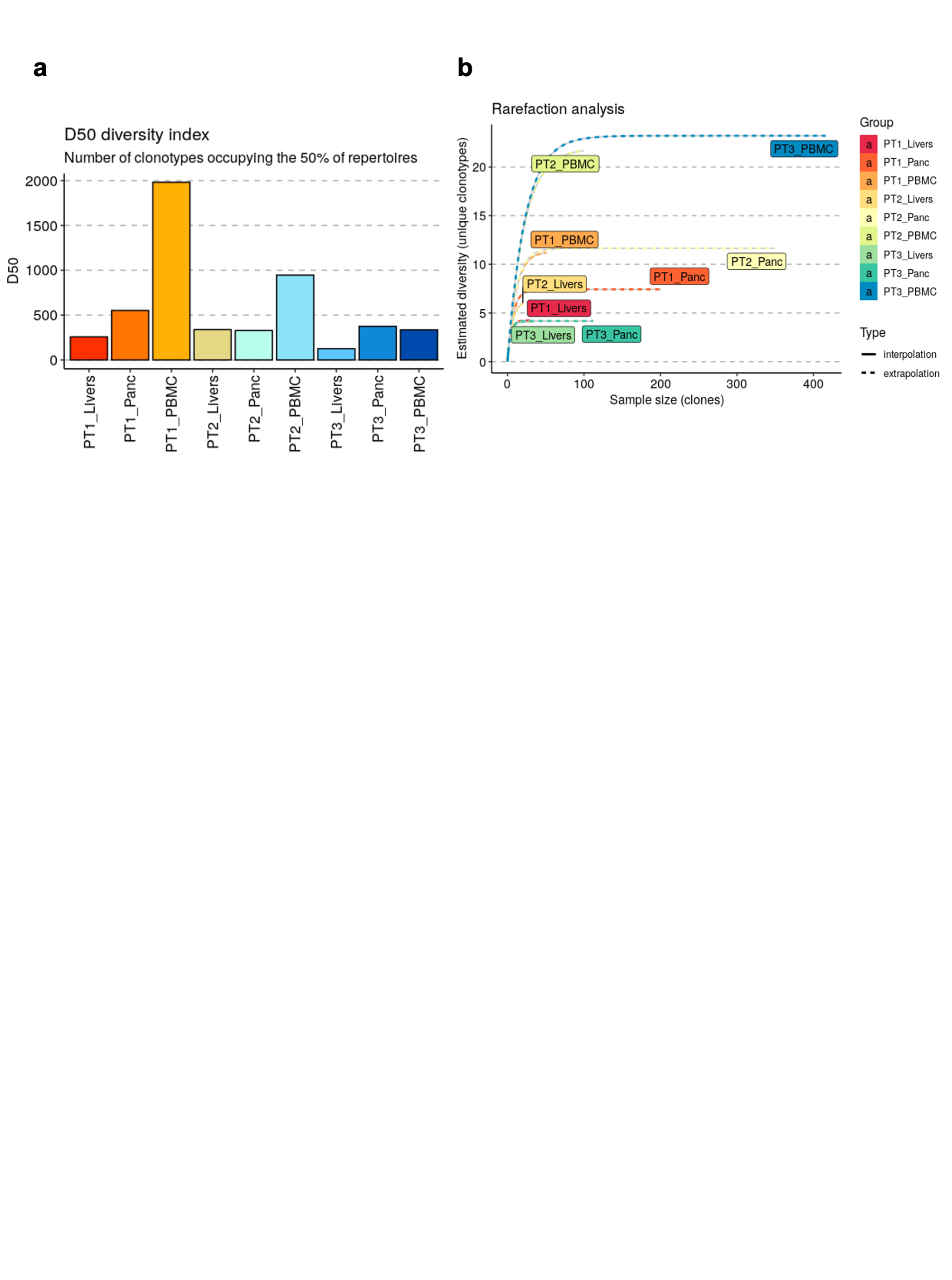

Supplement: Supplementary file 5 — Supplementary file5 (TIFF 17372 KB) [file 262_2025_4146_MOESM5_ESM.tiff]

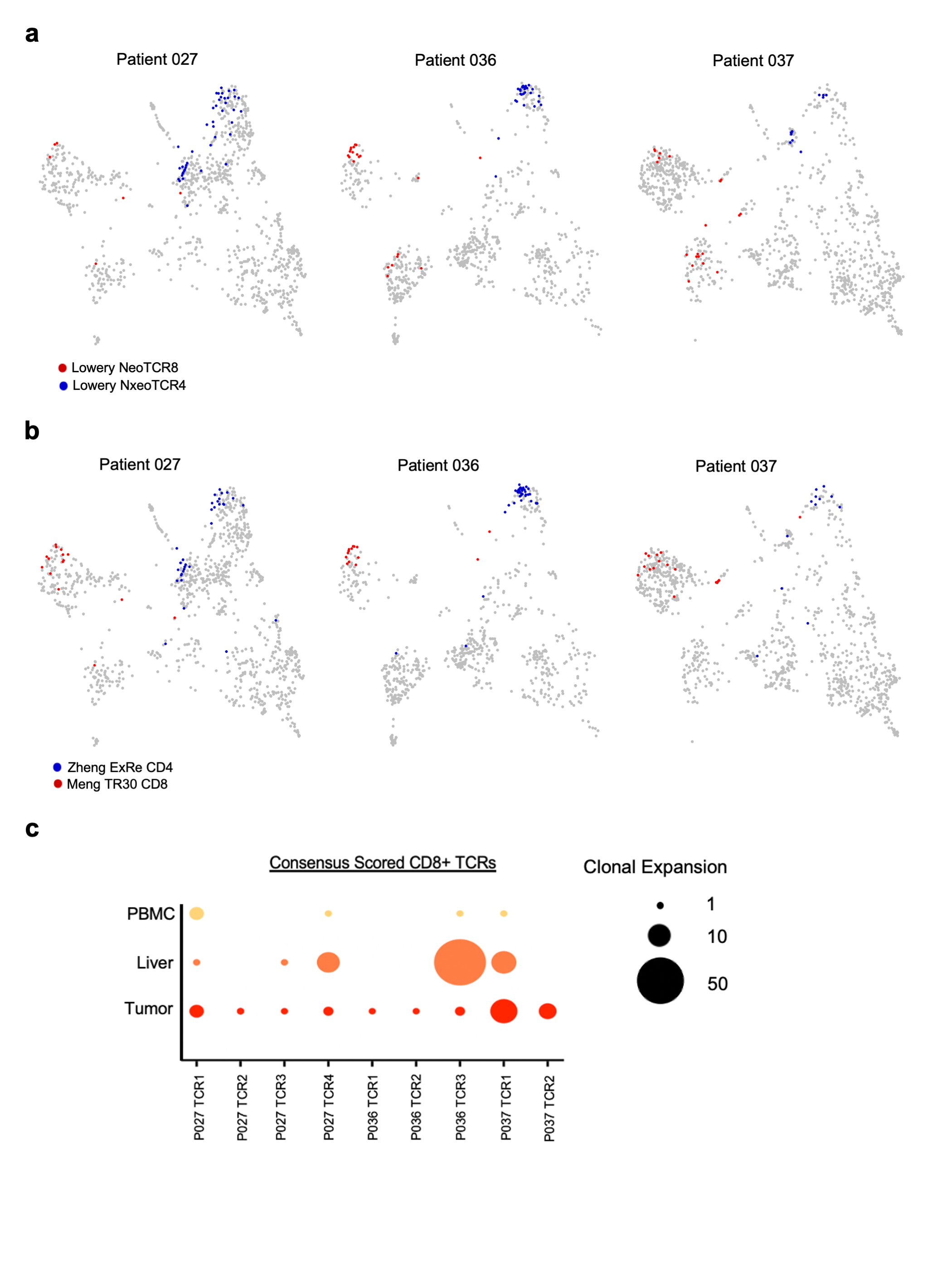

Supplement: Supplementary file 6 — Supplementary file6 (TIFF 17372 KB) [file 262_2025_4146_MOESM6_ESM.tiff]
