# Supplementary material for: A platform for multisite immune profiling of premetastatic pancreatic cancer at single-cell resolution
Source: Cancer Immunol Immunother. 2025 Aug 23;74(9):291. doi: 10.1007/s00262-025-04146-5 (PMC12374926; doi:10.1007/s00262-025-04146-5)
Supplement: Supplementary file 9 — Supplementary file9 (DOCX 19 KB) [file 262_2025_4146_MOESM9_ESM.docx]

**Supplementary Table S3. Patient Characteristics.**

|  | **Patient 027** | **Patient 036** | **Patient 037** |
| --- | --- | --- | --- |
| **Age at Diagnosis** | 66 | 66 | 61 |
| **Sex** | Female | Female | Female |
| **Location of Tumor** | Pancreatic Head | Pancreatic Head | Pancreatic Head |
| **NCCN Resectability Status** | Resectable | Resectable | Resectable |
| **Mutational Status** | - **KRAS** G12D - **CDKN2A/B** p16INK4a R58* and p14ARF P72L - **TET2** C1271fs*29 – subclonal - **TP53** R273C | - **KRAS** G12D - **ACVR1B** G448R – subclonal - **CDKN2A/B** p16INK4a Y44fs*9 - **TGFBR2** P129fs*3 - **TP53** V122fs*26 2 | Not tested |
| **Neoadjuvant Therapy** | None | None | None |
| **Ca 19-9 at Diagnosis** | 98 | 157 | 15 |
| **CEA at Diagnosis** | 7.4 | 2 | <1.73 |
| **Biliary Stent/Drainage** | Yes | Yes | Yes |
| **Bilirubin at Day of Surgery** | 5.6 | 1.4 | 1.1 |
| **Pathologic Stage** | T2N2 (Stage III) | T2N1 (Stage IIB) | T2N2 (Stage III) |
| **Resection Status** | R1 | R0 | R0 |
| **Histologic Grade** | G2, moderately differentiated | G2, moderately differentiated | G4, undifferentiated |
| **Lymphovascular Invasion** | Present | Present | Present |
| **Perineural Invasion** | Present | Present | Present |
| **Adjuvant Therapy** | mFOLFIRINOX | mFOLFIRINOX | mFOLFIRINOX |
| **Time to Radiographic Recurrence** | 7 months | N/A | 5 days |
| **Recurrence Type** | Hepatic | N/A | Hepatic |
| **Time to Last Follow Up** | 25 months | 27 months | 8 months |
| **Vital Status** | Dead | Alive | Dead |

**Supplementary Table S3. Clinical characteristics of enrolled patients.** The demographics, tumor characteristics, mutational status, pathologic stage, and disease status of the enrolled patients within this study.
